# *catena*-Poly[[bis­(1-ethyl-1*H*-imidazole-κ*N* ^3^)copper(II)]-μ-oxalato-κ^4^ *O* ^1^,*O* ^2^:*O* ^1′^,*O* ^2′^]

**DOI:** 10.1107/S1600536811043121

**Published:** 2011-10-22

**Authors:** Qian Xu

**Affiliations:** a Ordered Matter Science Research Center, College of Chemistry and Chemical Engineering, Southeast University, Nanjing 211189, People’s Republic of China

## Abstract

The title compound, [Cu(C_2_O_4_)(C_5_H_8_N_2_)_2_]_*n*_, is composed of one-dimensional linear chains running parallel to the *a* axis. In the chain, *trans*-[Cu(imidazole)_2_]^2+^ units are sequentially bridged by bis-bidentate oxalate ligands, resulting in an octa­hedral CuO_4_N_2_ donor set. The Cu⋯Cu separation through the oxalate bridge is 5.620 (5) Å. Both the Cu atoms and the C—C bond of the oxalate bridge are bis­ected by inversion centres.

## Related literature

For general background on ferroelectric organic compounds with framework structures, see: Fu *et al.* (2009 ▶); Ye *et al.* (2006 ▶); Zhang *et al.* (2008 ▶, 2010 ▶).
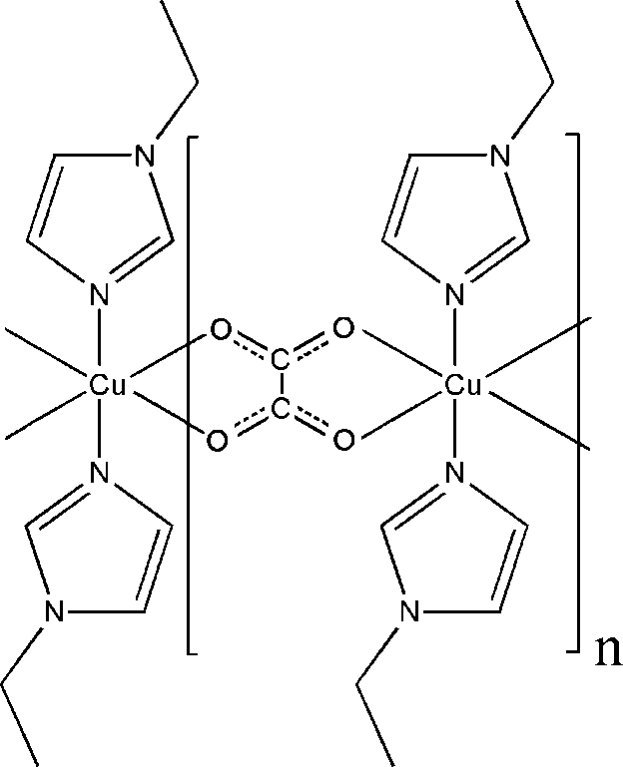


## Experimental

### 

#### Crystal data

[Cu(C_2_O_4_)(C_5_H_8_N_2_)_2_]
*M* *_r_* = 343.83
Monoclinic, 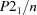

*a* = 5.6200 (11) Å
*b* = 8.8577 (18) Å
*c* = 14.481 (3) Å
β = 96.55 (3)°
*V* = 716.2 (2) Å^3^
*Z* = 2
Mo *K*α radiation
μ = 1.55 mm^−1^
*T* = 293 K
0.30 × 0.25 × 0.20 mm

#### Data collection

Rigaku SCXmini diffractometer
Absorption correction: multi-scan (*CrystalClear*; Rigaku, 2005 ▶) *T*_min_ = 0.635, *T*_max_ = 0.734
7300 measured reflections
1653 independent reflections
1267 reflections with *I* > 2σ(*I*)
*R*_int_ = 0.062

#### Refinement

*R*[*F* ^2^ > 2σ(*F* ^2^)] = 0.040
*wR*(*F* ^2^) = 0.092
*S* = 1.05
1653 reflections
98 parameters
H-atom parameters constrained
Δρ_max_ = 0.48 e Å^−3^
Δρ_min_ = −0.33 e Å^−3^

### 

Data collection: *CrystalClear* (Rigaku, 2005 ▶); cell refinement: *CrystalClear*; data reduction: *CrystalClear*; program(s) used to solve structure: *SHELXS97* (Sheldrick, 2008 ▶); program(s) used to refine structure: *SHELXL97* (Sheldrick, 2008 ▶); molecular graphics: *SHELXTL* (Sheldrick, 2008 ▶); software used to prepare material for publication: *SHELXL97*.

## Supplementary Material

Crystal structure: contains datablock(s) I, global. DOI: 10.1107/S1600536811043121/bg2425sup1.cif

Structure factors: contains datablock(s) I. DOI: 10.1107/S1600536811043121/bg2425Isup2.hkl

Additional supplementary materials:  crystallographic information; 3D view; checkCIF report

## Figures and Tables

**Fig. 1.**
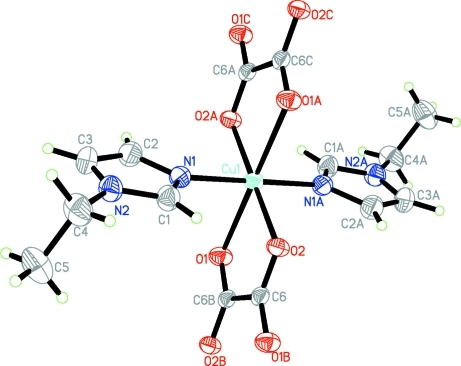
Ellipsoid plot of (I), ( 50% probability level). Symmetry codes A: -x, 1-y, 1-z. B: 1-x, 1-y, 1-z. C: -1+x, y, z.

**Fig. 2.**
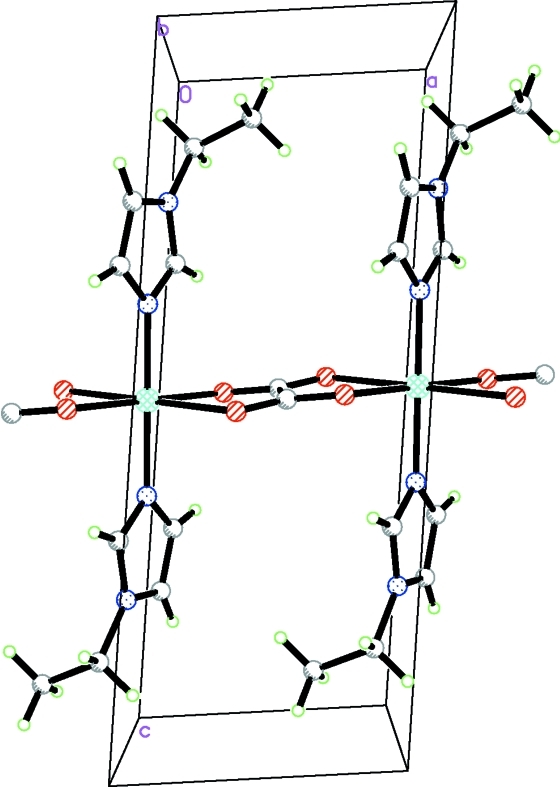
Packing diagram of the title compound showing the way in which chains are built up.

## supplementary crystallographic information

### Comment

As part of our ongoing study of potential ferroelectric materials we have determined the structure of the present copper complex and examined  its dielectric behaviour with temperature. This is the usual method for detecting these materials (Fu *et al.*, 2009; Ye *et al.*, 2006; Zhang *et al.*, 2008; Zhang *et al.*, 2010). Unfortunately, the dielectric constant for the title compound, Cu[C_5_H_8_N]_2_C_2_O_4_, (I) does not show any behavior indicating the onset of a ferroelectric phase change over the range 80 K to 298 K (m.p.319–329).

The Cu atoms are located on crystallographic inversion centers, and are coordinated to four oxygen atoms of two bridging oxalato ligands, also bisected by inversion centres, and two endocyclic nitrogen atoms from two crystallograhically related imidazole molecules, resulting in octahedral MO_4_N_2_ donor sets. Fig. 2 suggests the way in which oxalato-bridged chains build up. The Cu — Cu intrachain separation is 5.620 (5) Å.

### Experimental

A mixture of 1-ethyl imidazole (1.9 g, 20 mmol), cupric oxalate (1.5 g, 10 mmol) in water was stirred for several days at ambient temperature; blue block crystals were obtained on standing.

### Refinement

All H atoms were placed in geometrically idealized positions and constrained to ride on their parent atoms with C—H = 0.93–0.96 Å, and with *U*ĩso(H) = 1.2 *U*ĩso(C) or 1.5 Uĩso(C) for ethy H atoms..

### Crystal data

**Table:**

[Cu(C_2_O_4_)(C_5_H_8_N_2_)_2_]	*F*(000) = 354
*M**_r_* = 343.83	*D*_x_ = 1.594 Mg m^−^^3^
Monoclinic, *P*2_1_/*n*	Mo *K*α radiation, λ = 0.71073 Å
Hall symbol: -P 2yn	Cell parameters from 1653 reflections
*a* = 5.6200 (11) Å	θ = 2.3–27.5°
*b* = 8.8577 (18) Å	µ = 1.55 mm^−^^1^
*c* = 14.481 (3) Å	*T* = 293 K
β = 96.55 (3)°	Block, blue
*V* = 716.2 (2) Å^3^	0.30 × 0.25 × 0.20 mm
*Z* = 2	

### Data collection

**Table:**

Rigaku SCXmini diffractometer	1267 reflections with *I* > 2σ(*I*)
Radiation source: fine-focus sealed tube	*R*_int_ = 0.062
graphite	θ_max_ = 27.5°, θ_min_ = 3.7°
ω scans	*h* = −7→7
Absorption correction: multi-scan (*CrystalClear*; Rigaku, 2005)	*k* = −11→11
*T*_min_ = 0.635, *T*_max_ = 0.734	*l* = −18→18
7300 measured reflections	2 standard reflections every 150 reflections
1653 independent reflections	intensity decay: none

### Refinement

**Table:**

Refinement on *F*^2^	Primary atom site location: structure-invariant direct methods
Least-squares matrix: full	Secondary atom site location: difference Fourier map
*R*[*F*^2^ > 2σ(*F*^2^)] = 0.040	Hydrogen site location: inferred from neighbouring sites
*wR*(*F*^2^) = 0.092	H-atom parameters constrained
*S* = 1.05	*w* = 1/[σ^2^(*F*_o_^2^) + (0.0361*P*)^2^ + 0.3394*P*] where *P* = (*F*_o_^2^ + 2*F*_c_^2^)/3
1653 reflections	(Δ/σ)_max_ < 0.001
98 parameters	Δρ_max_ = 0.48 e Å^−^^3^
0 restraints	Δρ_min_ = −0.33 e Å^−^^3^

### Special details

**Table:**

Geometry. All e.s.d.'s (except the e.s.d. in the dihedral angle between two l.s. planes) are estimated using the full covariance matrix. The cell e.s.d.'s are taken into account individually in the estimation of e.s.d.'s in distances, angles and torsion angles; correlations between e.s.d.'s in cell parameters are only used when they are defined by crystal symmetry. An approximate (isotropic) treatment of cell e.s.d.'s is used for estimating e.s.d.'s involving l.s. planes.
Refinement. Refinement of *F*^2^ against ALL reflections. The weighted *R*-factor *wR* and goodness of fit *S* are based on *F*^2^, conventional *R*-factors *R* are based on *F*, with *F* set to zero for negative *F*^2^. The threshold expression of *F*^2^ > σ(*F*^2^) is used only for calculating *R*-factors(gt) *etc*. and is not relevant to the choice of reflections for refinement. *R*-factors based on *F*^2^ are statistically about twice as large as those based on *F*, and *R*- factors based on ALL data will be even larger.

### Fractional atomic coordinates and isotropic or equivalent isotropic displacement parameters (Å^2^)

**Table:**

	*x*	*y*	*z*	*U*_iso_*/*U*_eq_	
Cu1	0.0000	0.5000	0.5000	0.02341 (16)	
C1	0.0677 (5)	0.4366 (4)	0.3025 (2)	0.0326 (7)	
H1	0.1452	0.3463	0.3194	0.039*	
C2	−0.1151 (5)	0.6469 (4)	0.3090 (2)	0.0365 (7)	
H2	−0.1900	0.7304	0.3318	0.044*	
C3	−0.0853 (6)	0.6229 (4)	0.2184 (2)	0.0397 (8)	
H3	−0.1337	0.6861	0.1685	0.048*	
C4	0.1083 (6)	0.4132 (4)	0.1331 (2)	0.0439 (8)	
H4A	−0.0227	0.4136	0.0833	0.053*	
H4B	0.1477	0.3088	0.1482	0.053*	
C5	0.3213 (7)	0.4885 (4)	0.0999 (3)	0.0534 (9)	
H5A	0.2801	0.5899	0.0809	0.080*	
H5B	0.3701	0.4333	0.0482	0.080*	
H5C	0.4505	0.4906	0.1494	0.080*	
C6	0.4839 (4)	0.4123 (3)	0.49195 (17)	0.0210 (5)	
N1	−0.0177 (4)	0.5292 (3)	0.36168 (15)	0.0282 (6)	
N2	0.0296 (4)	0.4876 (3)	0.21516 (16)	0.0339 (6)	
O1	0.3372 (3)	0.6619 (2)	0.52150 (13)	0.0291 (5)	
O2	0.2754 (3)	0.3599 (2)	0.49398 (12)	0.0259 (4)	

### Atomic displacement parameters (Å^2^)

**Table:**

	*U*^11^	*U*^22^	*U*^33^	*U*^12^	*U*^13^	*U*^23^
Cu1	0.0188 (2)	0.0301 (3)	0.0218 (2)	0.0016 (2)	0.00409 (16)	−0.0002 (2)
C1	0.0357 (16)	0.0345 (16)	0.0279 (16)	0.0039 (14)	0.0056 (13)	0.0016 (13)
C2	0.0367 (16)	0.0403 (18)	0.0333 (16)	0.0065 (14)	0.0067 (13)	0.0027 (14)
C3	0.0414 (17)	0.0478 (19)	0.0291 (16)	0.0012 (16)	0.0010 (13)	0.0095 (15)
C4	0.050 (2)	0.056 (2)	0.0266 (16)	−0.0079 (17)	0.0082 (14)	−0.0087 (15)
C5	0.063 (2)	0.050 (2)	0.052 (2)	−0.0054 (19)	0.0277 (19)	0.0000 (18)
C6	0.0211 (12)	0.0244 (14)	0.0172 (12)	0.0034 (12)	0.0008 (10)	0.0010 (11)
N1	0.0265 (12)	0.0336 (15)	0.0248 (12)	0.0027 (10)	0.0048 (10)	0.0015 (10)
N2	0.0358 (13)	0.0422 (15)	0.0238 (12)	−0.0023 (12)	0.0042 (10)	−0.0010 (11)
O1	0.0235 (9)	0.0270 (11)	0.0374 (11)	0.0020 (8)	0.0069 (8)	−0.0030 (9)
O2	0.0197 (9)	0.0269 (10)	0.0316 (10)	−0.0014 (8)	0.0054 (8)	−0.0003 (9)

### Geometric parameters (Å, °)

**Table:**

Cu1—O2	1.9935 (18)	C3—H3	0.9300
Cu1—O2^i^	1.9935 (18)	C4—N2	1.470 (4)
Cu1—N1	2.011 (2)	C4—C5	1.497 (4)
Cu1—N1^i^	2.011 (2)	C4—H4A	0.9700
Cu1—O1^i^	2.3684 (18)	C4—H4B	0.9700
Cu1—O1	2.3684 (19)	C5—H5A	0.9600
C1—N1	1.316 (4)	C5—H5B	0.9600
C1—N2	1.337 (4)	C5—H5C	0.9600
C1—H1	0.9300	C6—O1^ii^	1.235 (3)
C2—C3	1.359 (4)	C6—O2	1.264 (3)
C2—N1	1.368 (4)	C6—C6^ii^	1.579 (5)
C2—H2	0.9300	O1—C6^ii^	1.235 (3)
C3—N2	1.365 (4)		
			
O2—Cu1—O2^i^	180.000 (1)	N2—C4—C5	112.7 (3)
O2—Cu1—N1	89.27 (8)	N2—C4—H4A	109.1
O2^i^—Cu1—N1	90.73 (8)	C5—C4—H4A	109.1
O2—Cu1—N1^i^	90.73 (8)	N2—C4—H4B	109.1
O2^i^—Cu1—N1^i^	89.27 (8)	C5—C4—H4B	109.1
N1—Cu1—N1^i^	180.000 (1)	H4A—C4—H4B	107.8
O2—Cu1—O1^i^	103.35 (7)	C4—C5—H5A	109.5
O2^i^—Cu1—O1^i^	76.65 (7)	C4—C5—H5B	109.5
N1—Cu1—O1^i^	89.94 (8)	H5A—C5—H5B	109.5
N1^i^—Cu1—O1^i^	90.06 (8)	C4—C5—H5C	109.5
O2—Cu1—O1	76.65 (7)	H5A—C5—H5C	109.5
O2^i^—Cu1—O1	103.35 (7)	H5B—C5—H5C	109.5
N1—Cu1—O1	90.06 (8)	O1^ii^—C6—O2	125.6 (2)
N1^i^—Cu1—O1	89.94 (8)	O1^ii^—C6—C6^ii^	117.7 (3)
O1^i^—Cu1—O1	180.00 (7)	O2—C6—C6^ii^	116.7 (3)
N1—C1—N2	112.0 (3)	C1—N1—C2	105.3 (2)
N1—C1—H1	124.0	C1—N1—Cu1	126.0 (2)
N2—C1—H1	124.0	C2—N1—Cu1	128.6 (2)
C3—C2—N1	109.5 (3)	C1—N2—C3	106.8 (2)
C3—C2—H2	125.2	C1—N2—C4	125.6 (3)
N1—C2—H2	125.2	C3—N2—C4	127.5 (3)
C2—C3—N2	106.3 (3)	C6^ii^—O1—Cu1	108.12 (16)
C2—C3—H3	126.8	C6—O2—Cu1	119.93 (16)
N2—C3—H3	126.8		

Symmetry codes: (i) −*x*, −*y*+1, −*z*+1; (ii) −*x*+1, −*y*+1, −*z*+1.
